# Hospitals in Baltimore

**Published:** 1880-05

**Authors:** 


HOSPITALS IN BALTIMORE.—In continuing the subject of hospitals in this city, it is obvious that only the more salient points can be referred to in the editorial department of the Journal. But it is hoped that such items may tend to awaken public attention to the subject generally, and evoke action calculated to mitigate, if not entirely to do away with or abolish such abuses and imperfections as may be found to exist in any of the institutions to which reference may be made.

*St. Joseph's* and the *Hebrew Hospitals* are in the eastern portion of the city, and are both admirably located and well constructed edifices, and we learn are well managed in the interests of the denominations to which they respectively belong, viz.: the Roman Catholic and Jewish churches; and, therefore, should they restrict their admissions to individuals reared in their respective communions, should receive the aid and countenance of the liberal and humane portion of our fellow-citizens.

*Johns Hopkins Hospital*, in the same part of the city, will receive attention as it approaches completion and enters upon its work.

*The Church Home*, in the same vicinity, is owned and mainly sustained by members of the Episcopal denomination in Baltimore, but is open to Christians of all or no denomination. A portion of the house is devoted to charity patients, or such as are unable to pay for care and attention during their confinement from accident or illness; and graded rates, according to location of apartments and other accommodations, are charged to others able and willing to pay for the same. Such patients may avail themselves of the services of the physicians and surgeons of the hospital staff, or select their own medical attendant, as they may choose. Religious instruction and services are held on the Sabbath day, open to all patients able and willing to participate in them, in conformity with the doctrines of evangelical Protestantism. The location, construction, hygienic surroundings and interior conveniences, nursing, culinary arrangements, sleeping apartments and skillful medical attendants conspire to render it an exceptionally desirable place for the sick and suffering. For one seeking rest from care, and in need of recuperation only, the admirable situation, fine views, etc., would render the Church Home a desirable retreat from the cares and perplexities of business life.

Situated in northwest Baltimore is another pleasantly located and excellently conducted hospital, the *Union Protestant Infirmary* or *Hospital*, on Division street. This hospital, as its name imports, is owned and under the care of the evangelical Protestant denominations of the city, and like the Church Home has its free and pay departments, and like it also patients may secure such rooms and other accommodations as their means or inclination may prompt them, and place themselves under the medical staff of the institution or select their own attendant, and remain as isolated or public as they may elect. The charges for private apartments, board and medical attendance need not exceed in amount good hotel board, and like other places receiving and caring for strangers, payments are only required once a week. Persons from a distance often engage quarters and board at this hospital, and employ their own medical attendant when they have a preference for a physician not connected with the institution.

The *University Hospital*, under the control of the Faculty of the University of Maryland, is pleasantly situated, and sufficiently retired and quiet for the ordinary purposes of a general hospital. It is well managed, so far as we have been able to learn, and has a staff of physicians and surgeons of skill and experience. It is also the Marine Hospital for the port of Baltimore. It is sufficiently removed from the lecture-rooms of the Faculty to prevent the unpleasant ideas and associations which close proximity to dissecting-rooms is almost certain to evoke in the mind of patients.

A very few words on the *City Hospital*, so called, will conclude what our space will allow to the subject in this issue of the Journal. It would be difficult, if not impossible, to find a more ineligible location for a city or, in fact, any other kind of hospital, in which to attempt the treatment of sick and wounded human beings, unable to provide or care for themselves, than this erroneously named concern; and we propose to show some of the reasons for this statement in an early issue of the Journal.

